# A General Procedure to Study Subcellular Models of Transsynaptic Signaling at Inhibitory Synapses

**DOI:** 10.3389/fninf.2016.00023

**Published:** 2016-06-30

**Authors:** Carmen A. Lupascu, Annunziato Morabito, Elisabetta Merenda, Silvia Marinelli, Cristina Marchetti, Rosanna Migliore, Enrico Cherubini, Michele Migliore

**Affiliations:** ^1^Institute of Biophysics, National Research CouncilPalermo, Italy; ^2^European Brain Research InstituteRome, Italy

**Keywords:** CA1 neurons, GABAergic synapses, gephyrin, fitting procedure, NEURON simulator, Python, Human Brain Project Collaboratory

## Abstract

Computational modeling of brain circuits requires the definition of many parameters that are difficult to determine from experimental findings. One way to help interpret these data is to fit them using a particular kinetic model. In this paper, we propose a general procedure to fit individual synaptic events recorded from voltage clamp experiments. Starting from any given model description (mod file) in the NEURON simulation environment, the procedure exploits user-defined constraints, dependencies, and rules for the parameters of the model to fit the time course of individual spontaneous synaptic events that are recorded experimentally. The procedure, implemented in NEURON, is currently available in ModelDB. A Python version is installed, and will be soon available for public use, as a standalone task in the Collaboratory Portal of the Human Brain Project. To illustrate the potential application of the procedure, we tested its use with various sets of experimental data on GABAergic synapses; gephyrin and gephyrin-dependent pathways were chosen as a suitable example of a kinetic model of synaptic transmission. For individual spontaneous inhibitory events in hippocampal pyramidal CA1 neurons, we found that gephyrin-dependent subcellular pathways may shape synaptic events at different levels, and can be correlated with cell- or event-specific activity history and/or pathological conditions.

## Introduction

The computational modeling of brain circuits, at practically any integration level, requires many parameters to be defined that, ideally, should be experimentally determined or constrained by experimental data or findings. However, many such parameters simply cannot be experimentally determined due to technical or conceptual limitations. In such cases, we may achieve better understanding of a neuronal function or mechanism by testing how and to what extent different theoretical models can take into account relevant experimental data. Fitting experimental data against a number of different models is a common way to do this (reviewed in Van Geit et al., 2008), and can help in the subsequent interpretation of the data. In general, experimental traces are fitted using specific approaches for specific purposes (e.g., Bekkers, 2003; Meisl et al., 2016). However, to the best of our knowledge, there is no easy, user-friendly, general procedure available for this purpose, especially in computational neuroscience. Our aim was thus to identify the appropriate conceptual structure of a procedure to obtain good, reliable fits of raw experimental traces of spontaneous synaptic events. This is an important step because spontaneous synaptic events have been so far exclusively analyzed using traces obtained by averaging many events. However, as can be easily imagined, each synapse in any given neuron has its own, independent, history of activation. The most likely physiological consequence is that the variables relative to the subcellular processes underlying synaptic transmission are different for each synapse. If a researcher is interested in testing a specific kinetic scheme implemented for specific biochemical pathways, the use of individual events is the most appropriate choice, since this approach would give information on the different combinations of model parameters that are consistent with the observed events. Averaging traces will lose a lot of relevant information.

We therefore present here the implementation of a procedure leading to the development of a unifying optimization method for individual synaptic events. Experimental data, kinetic models of synaptic transmission, and fitting parameters and their dependencies can be user defined/provided or gathered from databases. They can be used to generate optimized groups of parameters able to represent a population of synapses, either for simulation purposes or to study the functional consequences of a particular protein or subcellular synaptic transmission pathway. The procedure is implemented as a general tool that is available from the ModelDB depository (http://senselab.med.yale.edu/ModelDB/) for offline use, or within the Human Brain Project (HBP; Markram, 2012) infrastructure.

## Materials and methods

### Experimental procedure

#### Ethical approval

All experiments were performed in accordance with Directive 2010/63/EU of the European Parliament and of the Council on the protection of animals used for scientific purposes, and approved by both the Institute's Ethical Committee and the Italian authority veterinary service (Italian Ministry of Health). All efforts were made to avoid animal suffering and to minimize the number of animals used.

#### Hippocampal slice preparation

We obtained coronal hippocampal slices (300 μm thick) from juvenile (P21–30) C57Bl6/J male mice (three experiments, expA, expC, and expD) or from 3 to 4 months old wt (B6/SJL) or Tg2576 (carrying the Amyloid Precursor Protein KM670/671NL Swedish mutation) female mice (experiments expB and expE, respectively). Briefly, after being anesthetized with 2-Bromo-2-chloro-1,1,1-trifluoroethane the animals were decapitated. The brain was quickly removed from the skull and placed in ice-cold cutting solution containing: 126 mM choline, 17 mM glucose, 26 mM NaHCO_3_, 2.5 mM KCl, 1.4 mM NaH_2_PO_4_, 7 mM MgCl_2_, and 0.5 mM CaCl_2_(equilibrated with 95% O_2_/5% CO_2_). We cut coronal slices with a vibratome and stored them for 45 min at 32°C and thereafter at room temperature (22–24°C). Individual slices were transferred to a recording chamber where they were continuously superfused with an oxygenated artificial cerebrospinal fluid (ACSF) (95% O_2_ and 5% CO_2_), at a rate of 3 ml/min, containing 126 mM NaCl, 2.5 mM KCl, 2 mM CaCl_2_, 2 mM MgSO_4_, 1.25 mM NaH_2_PO_4_, 26 mM NaHCO_3_, and 10 mM glucose (pH 7.4).

#### Electrophysiological recordings

From the coronal slices, we obtained whole-cell voltage clamp recordings from CA1 pyramidal neurons (PNs) at 32°C under infrared differential interference contrast imaging with borosilicate glass capillaries, which had resistances between 3 and 4 MΩ. We made voltage-clamp recordings of GABA_A_- mediated spontaneous inhibitory post-synaptic currents (sIPSCs) in the presence of the AMPA receptor antagonist 6,7-Dinitroquinoxaline-2,3-dione (DNQX 20 μM) and the NMDA receptor antagonist D-(-)-2-Amino-5-phosphonopentanoic acid (D-AP5 50 μM). The recording electrodes were filled with the following intracellular solution: 70 mM KGluconate, 70 mM KCl, 70 mM HEPES, 10 mM EGTA, 1 mM MgCl_2_, 4 mM MgATP, and 3 mM Na_2_GTP. The intracellular chloride concentration was dependent on the intracellular solution of the pipette, which yielded a calculated reversal potential for chloride of ~ −16 mV. PNs were held at −70 mV (C57Bl6/J mice) or −75 mV (B6/SJL and Tg2576 mice); GABA-mediated events were therefore detected as inward currents. Series resistance (Rs) was monitored throughout the experiment and recordings were discarded if Rs changed by 25% of its initial value. The data obtained were then used to test the performance of the fitting procedure. To this end, data were arbitrarily divided in 5 different experiments (expA-E).

### Computational procedure

We carried out all simulations using an integrated NEURON (v7.4, Carnevale and Hines, 2006) and Python (v2.7.10, Hines et al., 2009) parallel code on different systems, including a multiprocessor desktop Windows PC and a large supercomputer (IBM BlueGene/Q, FERMI machine, Cineca, Italy). The model and simulation files can be downloaded from the ModelDB database (http://senselab.med.yale.edu/ModelDB/, a.n. 182129); the HBP standalone task and jupyter notebook can be accessed from the Collaboratory Portal (https://collab.humanbrainproject.eu/#/collab/704/).

For the optimization, the NEURON built-in PRAXIS principal axis method for minimizing a function was used. The parallel implementation used the NEURON's ParallelContext() class with a bulletin board style analogous to LINDA. The Python standalone version requires task-sdk, a set of tools and libraries to develop code that can be registered, shared, launched and tracked using the HBP Platform. The public HBP Collab is implemented as a jupyter notebook, and allows community use, development, and improvements. The graphical interface of the jupyter notebook version is implemented using standard packages as plotly and ipywidgets. More details on the design and architecture of the procedure itself are presented and discussed in Results.

For the biophysical implementation of the cells, we used a simple single compartment (10 μm in diameter and length) with passive properties commonly used for CA1 PNs (*Cm* = 1μF/cm^2^, *Rm* = 28,000 Ω/cm^2^) and a resting potential set to the voltage clamp value these cells were held at in the experiments (−70 or −75 mV). However, to test for possible systematic errors, we also ran additional simulations using a morphologically and biophysically accurate model (cell 5038804, from ModelDB a.c.55035, Migliore et al., 2005). Of the many methods, error cost functions, and algorithms that can be used to fit electrophysiological experimental traces (Van Geit et al., 2008), we used the built-in NEURON principal axis steepest descent method (Praxis). In preliminary simulations we also used the new, open source, Distributed Evolutionary Algorithms in Python (DEAP).

To test our procedure with a set of experimental traces, we first needed to implement an appropriate kinetic model of synaptic transmission. The classic and widely used bi-exponential synaptic conductance change does not capture well the resulting variabilities of the IPSCs. Most importantly, however, it cannot give much information on the subcellular pathways underlying synaptic transmission. We were interested in testing a model for at least one of the biochemical pathways involving at least one of the tens of thousands of proteins present in a synapse. We choose to implement a relatively basic model for gephyrin. There are three main reasons for this choice: (1) it is one of the major players for synaptic transmission, and of particular importance in inhibitory synapses (reviewed in Choii and Ko, 2015) (2) we were able to implement a specific kinetic model simple enough to be, at the same time, informative and analytically solvable, and (3) we plan to carry out soon (for a future paper) experiments using a new type of antibody to selectively block Gephyrin *ex vivo*, to compare experimental data with the model's prediction.

## Results

**Figure 1:**
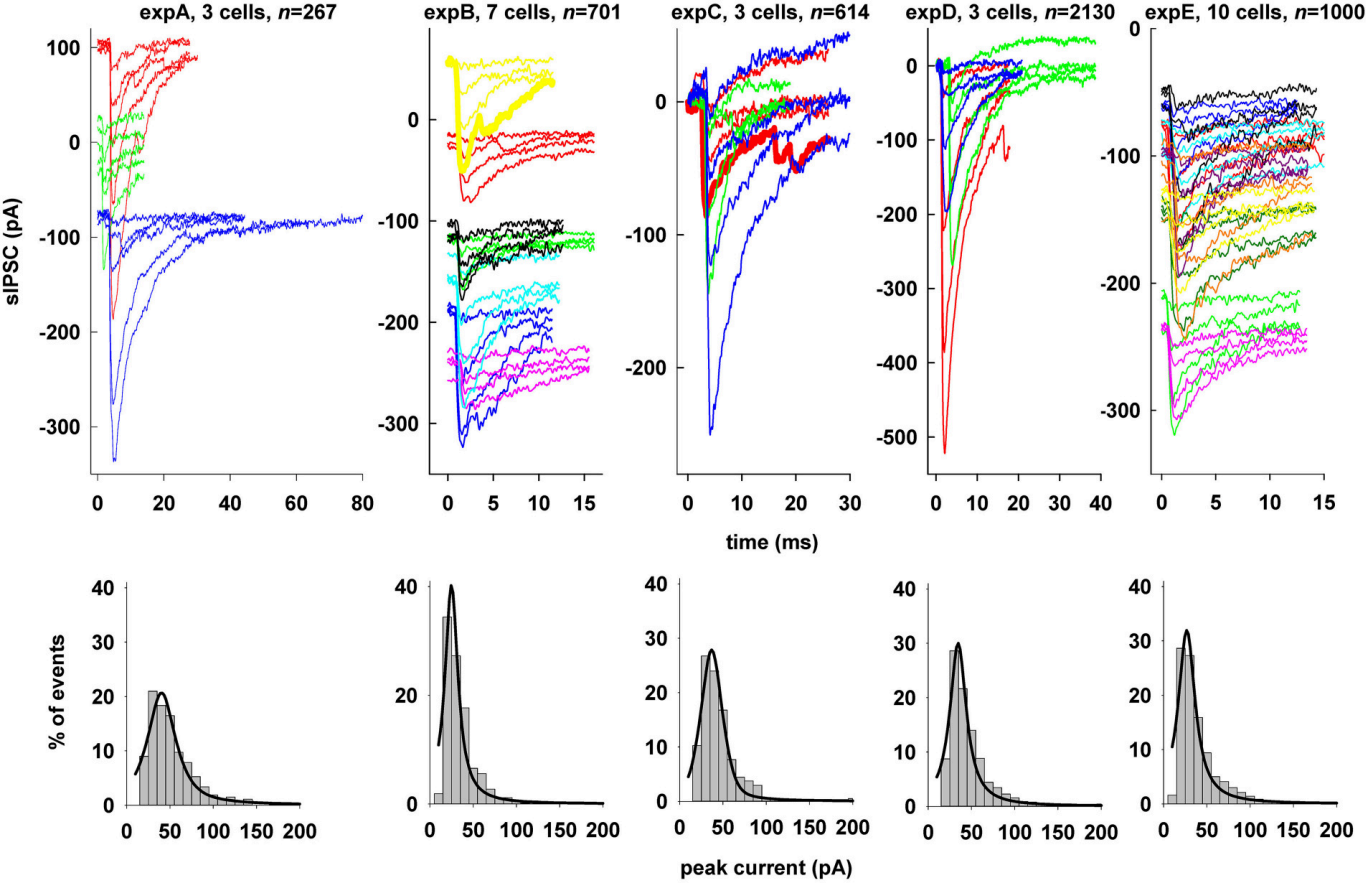
**Spontaneous inhibitory synaptic currents on hippocampal CA1 pyramidal neurons. Top:** Representative raw experimental traces of spontaneous events recorded from the five different experiments used; thick lines highlight examples of traces with multiple events; **Bottom:** distribution of peak sIPSCs (gray bars), from raw data. The distributions were fitted by a Pseudo-Voigt, 4-parameter equation:
$f\;=\;a\;\cdot \;\left(\frac{c}{1\;+\;{\left(\frac{x\;-\;x_{0}}{b}\right)}^{2}}\;+\;\left(1\;-\;c\right)\;\cdot \;e^{-0.5\cdot {\left(\frac{x\;-\;x_{0}}{b}\right)}^{2}}\right)$ The parameters best fitting each experiment were: *expA*, *a* = 0.2069, *b* = 18.7436, *c* = 0.8087, *x*_0_ = 40.2020 (*R* = 0.9681); *expB*, *a* = 0.4024, *b* = 9.0292, *c* = 1.0000, *x*_0_ = 24.8492 (*R* = 0.9602); *expC*, *a* = 0.2785, *b* = 13.3410, *c* = 0.4665, *x*_0_ = 36.9253 (*R* = 0.9811); *expD*, *a* = 0.2998, *b* = 11.5380, *c* = 1.0000, *x*_0_ = 34.6231 (*R* = 0.9715); *expE*, *a* = 0.3193, *b* = 11.7930, *c* = 1.0000, *x*_0_ = 26.8240 (*R* = 0.9633).

From five different experiments, we obtained a total of 4712 raw experimental sIPSC recordings to use in our fitting procedure. Representative sIPSC traces from each of the five experiments can be seen in Figure 1 (top), showing the relatively wide range of peak currents and duration obtained. For the purpose of this work, we also included traces that show multiple events (thick traces in Figure 1, expB and expC) or that are clearly unsuitable for fitting due to inadequate amplitude and/or large background noise. For all experiments, the distribution of peak currents was well approximated from the raw data by a 4-parameter pseudo-Voigt distribution (Figure 1, bottom). Approximately 25–30% of the events exhibited a peak current in the range of 20–30 pA, 2–3 times larger than GABAergic spontaneous events recorded from CA1 PNs of adult Wistar rats (Cossart et al., 2000).

**Figure 2:**
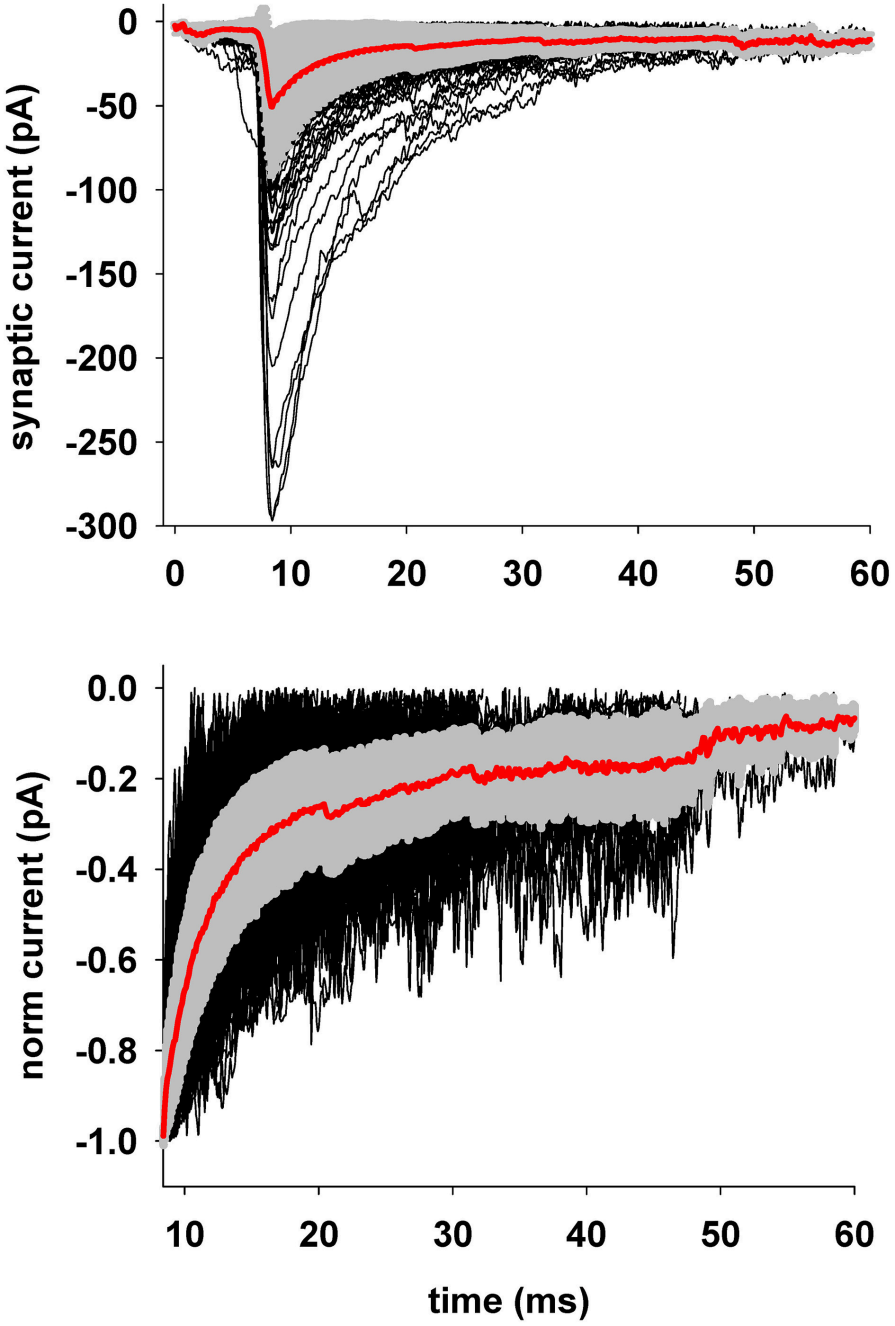
**Preprocessing of experimental traces before fitting. Top:** The plot shows the average (±sd) of all experimental traces selected for fitting, after preprocessing to isolate single events and remove holding and baseline currents; **Bottom:** to highlight the variability in the decay time, traces are shown aligned and normalized to the peak current; for clarity only the decay time course is shown.

To illustrate the typical range of basic properties of the events, we plotted all traces from expC (after baseline removal) aligned with respect to their peak time (Figure 2, top) and normalized with respect to their peak value (Figure 2, bottom). A comparison between the average curve (Figure 2, thick red lines) and the traces outside the standard deviation region (Figure 2, gray areas around the average) reveals the wide range of values that can be recorded.

**Figure 3:**
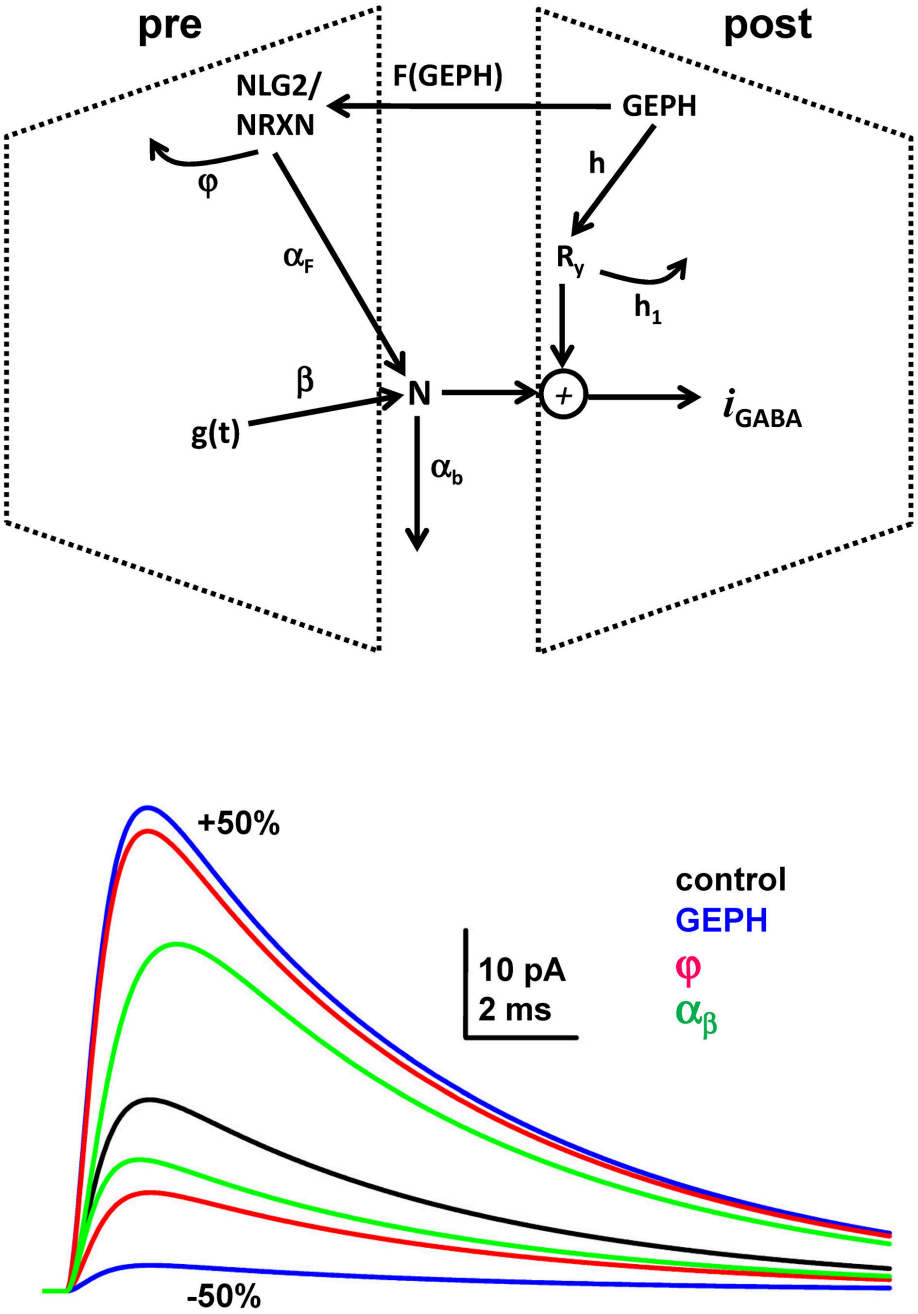
**The synaptic transmission scheme used for all simulations. Top:** The scheme represents the specific pre and post-synaptic subcellular processes that we wished to investigate (see main text for explanation); **Bottom:** somatic current (calculated from the analytical solution of the equations describing the synaptic transmission scheme) during a single synaptic activation under control conditions (black trace), and after 50% increase/decrease of GEPH, φ, and α_b_; we used the parameter values obtained under control conditions for the best fit.

For this work, we implemented a relatively simple subcellular transsynaptic inhibitory signaling pathway including pre- and post-synaptic scaffolding proteins (Figure 3). In this kinetic scheme, the inhibitory current, *I*_*GABAA*_, elicited by synaptic activation, is modulated by the following variables (in arbitrary units, unless specified otherwise):

- gephyrin clusters (GEPH)
- Neuroligin/Neurexin clusters (NLG2/NRXN)
- Neurotransmitter molecules (N)
- Postsynaptic receptors (Ry)
- Basic time course of synaptic conductance (*g(t)*)

Several rate constants form the set of fitting parameters and are responsible for the dynamics of the overall synaptic current. The overall kinetic scheme is implemented as a perturbation of *g(t)*, modeled using the phenomenological model of neocortical synapses introduced and discussed by Tsodyks and Markram (1997) and Abbott et al. (1997):

$$g(t)=w\;\cdot \;\left[\exp\left(-\frac{t}{\tau_{d}}\right)-\exp\left(-\frac{t}{\tau_{r}}\right)\right]$$

where *w* is the peak synaptic conductance, τ_*r*_ and τ_*d*_ are the rise and decay time constant, respectively. In the absence of this additional subcellular pathway (Figure 3, top), it would generate the typical double exponential time course. With the addition of the mechanisms represented in Figure 3, the set of differential equations describing the synaptic current dynamics is:

$$\frac{dN}{dt}=\beta \;\cdot \;\alpha_{f}\;\cdot \;g(t)\;\cdot \;\mathrm{NLG}2-\alpha_{b}\;\cdot \;N$$

$$\frac{dNLG2}{dt}=\frac{Nl_{max}\;\cdot \text{ F}(\mathrm{GEPH})}{1\;+\;N_{1/2}/\mathrm{NLG}2}-\varphi \;\cdot \;\mathrm{NLG}2$$

$$\frac{dR_{y}}{dt}=h\;\cdot \;\mathrm{GEPH}-\;h_{1}\;\cdot \;R_{y}$$

with the synaptic current defined as:

$$I_{\mathrm{GABAA}}=c_{1}\;\cdot \;N\;\cdot \;R_{y}\;\cdot \;(v-e_{rev})$$

where *c*_1_ is a constant, *v* the membrane potential and *e*_*rev*_ the reversal potential. We assumed that *GEPH* is constant throughout the duration of a synaptic event, that the peak value of *NLG2* depends on *GEPH*, and that it changes according to simple Michaelis-Menten kinetics, with *F*(*GEPH*) = 1. We preferred this minimal formulation over others that use more or less variables and dynamics, because we considered it sufficient to capture the overall effects of gephyrin and because it has several important properties for our purposes. For example, the inclusion of *h* and *h*_1_ means that it is easier to separate the effects caused by additional modulation of the postsynaptic response through GEPH-dependent mechanisms, without interfering with presynaptic pathways. Another extremely useful feature of this implementation is that the system permits an analytical solution; assuming a simple relationship between *GEPH* and *NLG2* as

$$Nl_{max}=GEPH,\;N_{half}=GEPH/2$$

the general solution for the variables of the system is then

$$\begin{array}{l} \mathrm{NLG}2=\frac{GEPH-GEPH\;\cdot \;\varphi }{2\varphi },\;R_{y}=\frac{h}{h_{1}}\;\cdot \;\mathrm{GEPH}\\ N(t)={\text{e}}^{-\alpha_{b}\cdot t}\;\cdot \;\{C[1]+\beta \;\cdot \;\alpha_{f}\;\cdot \text{ NLG}2\;\cdot \;w\cdot \\ \;\left(\frac{{\text{e}}^{(\alpha_{b}-\frac{1}{\tau_{D}})\cdot t}}{1-\alpha_{b}\tau_{D}}-\frac{{\text{e}}^{(\alpha_{b}-\frac{1}{\tau_{R}})\cdot t}}{1-\alpha_{b}\tau_{R}}\right)\} \end{array}$$

where *C*[1] depends on the initial conditions. Assuming the initial condition *N(0)* = *0*, the current *I*_*GABAA*_ will be

(1) $$I_{GABAA}=I_{FACT}\frac{\left[\left(1-\alpha_{b}\tau_{D}\right)-\left(1-\alpha_{b}\tau_{R}\right)\right]\;\cdot \;e^{-\alpha_{b}\cdot t}+\left(1-\alpha_{b}\tau_{R}\right)\;\cdot \;e^{-\frac{t}{\tau_{D}}}-(1-\alpha_{b}\tau_{D})\;\cdot \;e^{-\frac{t}{\tau_{R}}}}{\left(1-\alpha_{b}\tau_{D})\;\cdot \;(1-\alpha_{b}\tau_{R}\right)}\;\left(v-e_{GABAA}\right)$$

where

(2) $$I_{FACT}=c_{1}\;\cdot \;\frac{h}{h_{1}}\;\cdot \;\left[\frac{2\;\cdot \;\mathrm{GEP}H^{2}-\varphi \;\cdot \;\mathrm{GEP}H^{2}}{2\;\cdot \;\varphi }\right]\;.\beta \;\cdot \;\alpha_{f}\;\cdot \;w$$

The overall synaptic current will then be mainly dominated by: (1) the number of GEPH, squared, involved on the postsynaptic side; (2) the hyperbolic dependence from the NLG2/NRXN turnover rate, φ; and, (3) the rate at which released neurotransmitter molecules diffuse away from the synaptic cleft, α_*b*_ The effect of a 50% change in these parameters is shown on the graph on Figure 3, and demonstrates the larger overall consequence caused by an increase or decrease of GEPH (Figure 3 graph, blue lines), with respect to the other synaptic parameters. These results illustrate the fundamental role of a stable synaptic scaffolding mechanism for a reliable transsynaptic signal transmission, which can be easily disrupted by small changes in GEPH caused by pathological conditions. We used this model to fit all the experimental traces.

**Figure 4:**
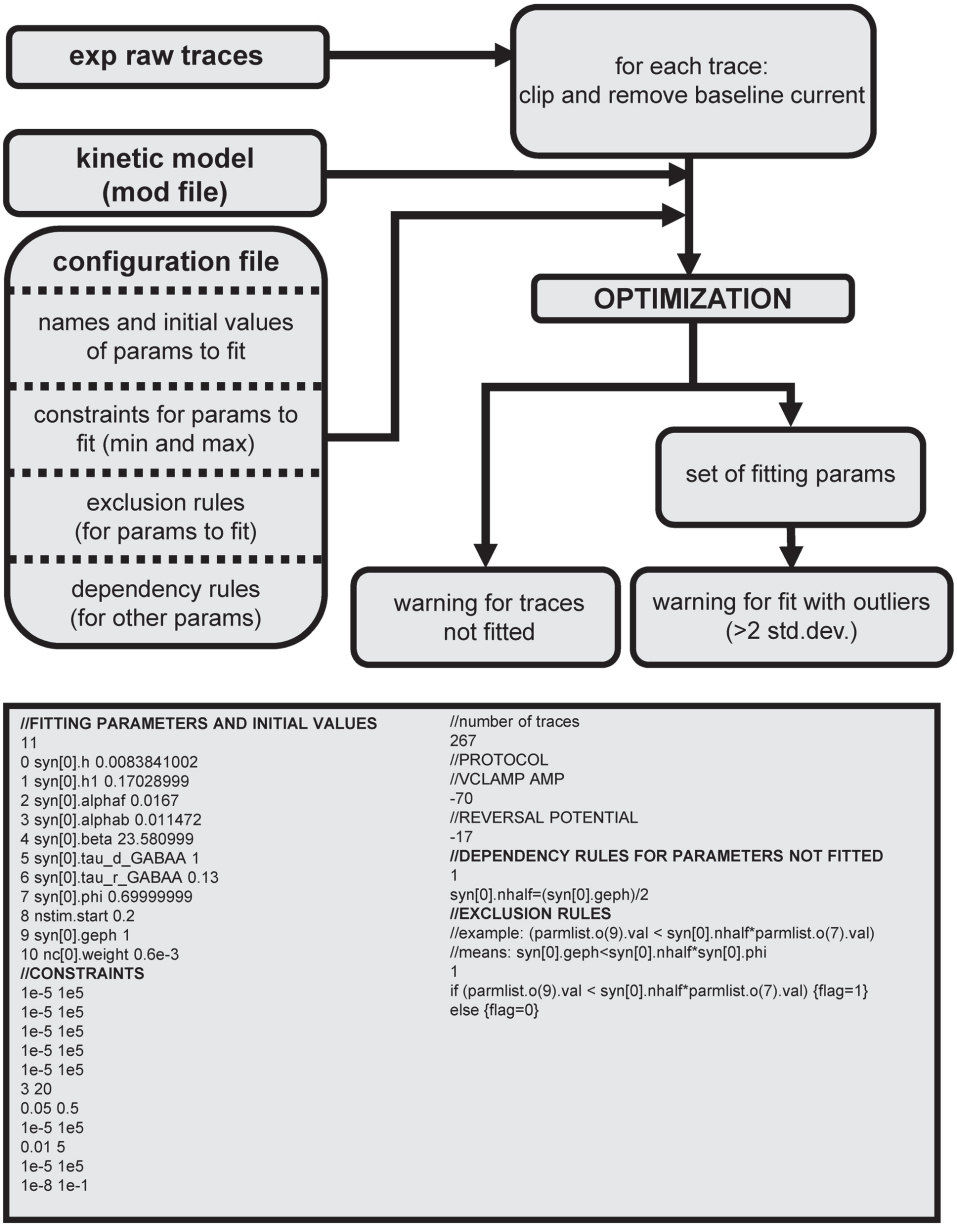
**Schematic organization of the general fitting procedure and a typical configuration file**.

The fitting procedure (Figure 4, top), was implemented in NEURON and Python. A pseudocode illustrating its extremely simple architecture is presented below. Traces were initially preprocessed to remove baseline current, and clipped to avoid overlapping synaptic activations that could significantly distort the time course of an individual event. To do this, the program identified relatively large current changes (more than 10% of the peak current), found using a moving time window that was dynamically determined for each trace. In addition, to avoid contamination by smaller events during the late phase of the current decay, traces were clipped when the current decayed to 20% of the maximum value. A user-defined configuration file provides all the information needed to fit the traces with a given synaptic kinetic model, with equations implemented in the usual NEURON model description language (i.e., in a “mod” file). The configuration file includes the name of the parameters to be fitted, their allowed range of variation, exclusion rules, and an optional set of dependencies for other parameters. A typical file is shown in Figure 4 (bottom). The definition of the relevant parameters and experimental protocol in a configuration file makes the procedure independent from the specific kinetic model, and enhances generality and usability. Every block inside the file is commented out to illustrate the kind of information needed to configure the fitting process. We tried to keep to a minimum the number of user-defined parameters. They can be eventually expanded according to the user community requests to include more technical parameters, such as number of iterations or random initial conditions, error threshold, parameters for trace extraction, etc. In preliminary tests, we explored different cost functions considering general features, such as the trace width when the current decayed to 10, 50, or 90% of the maximum. However, we found this approach unreliable due to the level of noise in most traces. We thus chose to minimize the classic root mean squared error (RMSE) between the time course of the experimental and simulated currents. An average RMSE lower than 10% of the peak current was chosen as the acceptable threshold for fit. We performed preliminary tests to find a reasonable number of randomized initial conditions and iteration steps, in terms of computational time and quality of fits, and found that a set of 100 initial conditions (uniformly randomized within a user-defined range) and up to 3000 iteration steps were sufficient; larger sets did not result in any qualitative improvement of the fits.

**Table:** 

1. load config and mod files
2. load experimental traces
3. **for** i = 1 to number of traces **do**
4. preprocess trace (returns trace length)
5. **if** trace length >= *minimum length* **then**
6. **for** j = 1 to number of initial configurations **do**
7. randomize initial parameters
8. Praxis optimization (NEURON simulations, return RMSE)
9. **if** RMSE < maximum tolerated error **then**
10. write ensemble of optimized parameters
11. **end if**
12. **end for**
13. **end if**
14. **end for**

To better explore the parameter space, each attempt to fit a trace began with a randomized set of initial conditions for all parameters (according to the relative range defined in the configuration file) and proceeded until reaching either convergence or the maximum number of iterations. Under these conditions, each fit attempt required an average of ≈2000 iteration steps, corresponding to ≈940,000,000 independent simulations to fit all 4712 experimental traces. This process is ideally suited for parallel processing. On an IBM BlueGene/Q using 2048 processors each fit took, on average, 1.5 s of CPU time. At the end of the procedure, the ensemble of parameters fitting each experimental trace was saved for further use and analysis. Warning messages were reported in a file for traces that either could not be fitted (i.e., with an RMSE >10% of the maximum current), or where the RMSE was within the threshold but one or more parameters showed an outlier value. About 38% of the traces were excluded for these problems. Considering that we are using non-averaged raw traces we think that this proportion is not too restrictive. Most of the rejected traces (25%) were excluded because of the error threshold, and the rest (13%) were not fitted because the semiautomatic trace selection process found them too short (< 5 ms after peak time), or overlapping with another event before they returned to an amplitude below 20% of the peak. The error threshold and the number of iterations may change the rejection rate. However, raising the error threshold would not be a good choice, because it may accept fits that do not represent well the kinetic model at study. Increasing the number of iterations could also reduce the rejection rate but it would require more computational time. This is part of the classic trade-off between computational time and fitting accuracy.

It can also be argued that a cost function based on the running mean rather than directly on the raw data might give better results. To test this hypothesis, the running mean was used to carry out the optimization for the 267 traces from expA. We found that more than 50% of the events that were accepted using the raw data were rejected using the running mean, and only 3.74% of those that were rejected were now accepted. One of the main reason for this low performance of the running mean may be that it tends to weight more the part of the curve around the peak.

**Figure 5:**
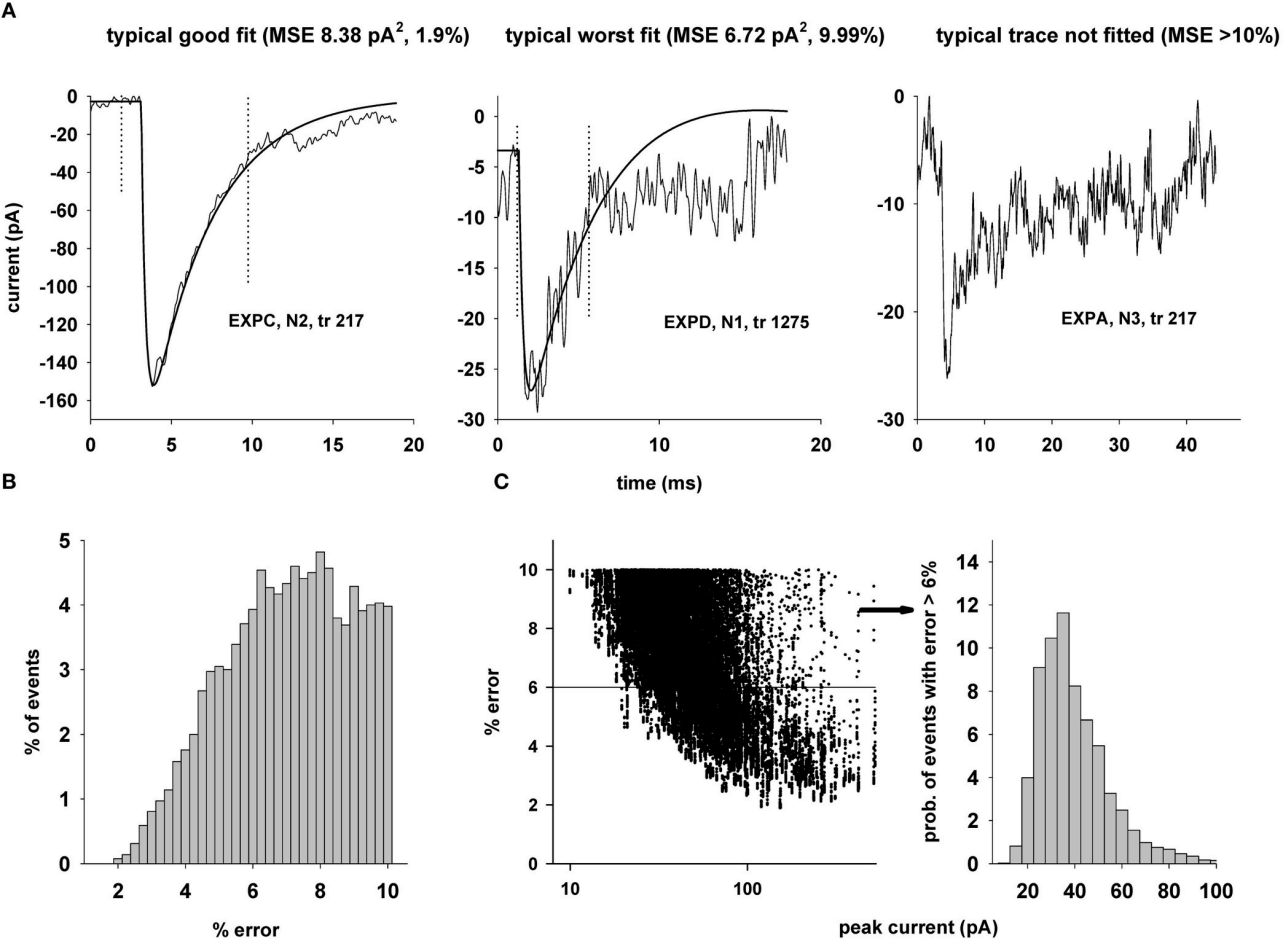
**Typical results of the fitting procedure. (A)** The plots show experimental traces and one fit for typical cases of good (left) and worst (middle) fit, and one of the traces for which a reasonable fit could not be found; **(B)** Proportion of events with a given fit error; **(C)** (left) Distribution of fit error as a function of the peak current; (right) distribution of peak current for traces with a fit error >6%.

Typical examples of traces and fit are plotted in Figure 5A. Larger events resulted in fits with below average RMSE (Figure 5A, left). However, even relatively small events can be reasonably fitted. As shown in Figure 5A (middle), clipping the raw trace when it decayed to 80% of the peak was especially useful in these cases (Figure 5A, dotted vertical lines). For many small events the background noise was too strong to obtain a reasonable fit (Figure 5A, right). Overall, a best fit was obtained for 2925 traces, and the error distribution for these cases (Figure 5B) reveals a substantial proportion of events (≈68%) with an error rather uniformly distributed in the range 6–10%. The distribution as a function of the peak current (Figure 5C) shows that this occurred for events in the range of 25–35 pA in many cases.

To test the performance of the DEAP approach, we carried out test simulations using all 267 traces from expA (see Figure 5A). Preliminary simulations showed that we could obtain fits with an RMSE relatively close to that obtained with the NEURON built-in Praxis method, using a population of 300 individuals and at least 60 generations. On a Linux cluster, using 320 processors, a fit of a single trace took ≈59,500 s. Using 2048 processors, 100 fit attempts would therefore take ≈930,000 s, i.e., more than 250 h to fit all 267 traces. The same process took less than 2 h using the Praxis method on a BlueGene/Q using 2048 processors. We therefore decided not to use the DEAP algorithm in this case.

To demonstrate the flexibility, applicability, and ease of use of the software, we used it to fit the traces from expA with another model. We chose the simple double-exponential kinetic scheme built-in in the NEURON simulation environment. This was done by modifying only the configuration file as shown below:

//FITTING PARAMETERS AND INITIAL VALUES

4

0 syn[0].tau1 1.6

1 syn[0].tau2 7.7

2 nstim.start 0.2

3 nc[0].weight 1.2e-3

//CONSTRAINTS

0 2

2 10

1e-5 1e5

1e-5 1e5

//DEPENDENCY RULES FOR PARAMETERS NOT FITTED

0

//EXCLUSION RULES

//example: (parmlist.o(9).val < syn[0].nhalf^*^parmlist.o(7).val)

//means: syn[0].geph < syn[0].nhalf^*^syn[0].phi

0

//PARAMETERS WARNING (ALL EXCEPT)

0

In comparison with our model, less traces were fitted with an error below 10% (77.15% instead of 77.90%) producing less ensembles of optimized parameters (8578 instead of 10,152). Also, the error distribution (not shown) contained a bigger proportion of events (65 vs. 55%) with an error in the upper range (6–10%).

**Figure 6:**
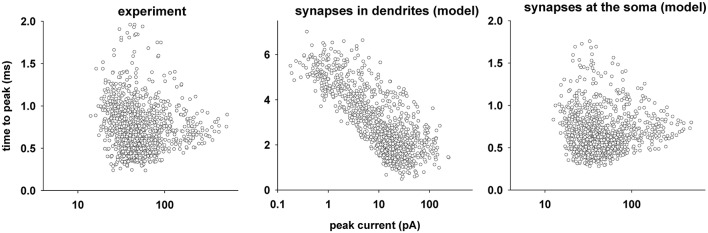
**Space clamp error. Left:** Time to peak of synaptic current as a function of the peak, from expD experimental traces that were selected for fitting; **Middle:** time to peak of synaptic current as a function of its peak, from simulations in which a synapse with a random parameter ensemble was placed at a random dendritic location; **Right:** time to peak of synaptic current as a function of its peak, from simulations in which all simulations carried out for the middle panel were repeated with the synapse in the soma.

The next step was to figure out if the fit results are a reasonable representation of the real synapses. We thus decided to use the component of time to peak current to test if the fits obtained with our procedure are affected by alterations in the time course of a synaptic current due to inadequate voltage clamp of dendrites or filtering properties of the membrane. For this purpose, we began from the results obtained in ExpD and selected all experimental traces for which it was possible to obtain a fit. We selected 1240 suitable traces from a total of 2130; the experimental values of the time to peak are plotted in Figure 6 (left). They show a relatively compact range of peak currents, with time to peak values in the range 0.2–2 ms and no correlation with peak current values (Pearson's correlation, *p* > 0.05). To assess whether these values are consistent with synapses located in the dendrites or at the soma, we performed simulations using the morphologically accurate CA1 neuron; we used 1112 synapses (two for each of the 556 dendritic membrane segments of the neuron) to generate single synaptic events, each with an ensemble of parameters randomly chosen among those obtained for the best fits of the experimental traces. The results for the time to peak of each event are plotted in Figure 6 (middle). A wide range of values in the range of 0.4–7 ms is spread over an even wider range of measured peak current values, inversely correlated with the distance of the synapse from the soma (Pearson's correlation −0.451, *p* < 0.001), and consistent with what can be expected by the filtering properties of the membrane. We then repeated the same set of simulations with a synapse placed at the soma. The results (Figure 6, right) show a distribution similar to the experimental values, with no correlation with the peak current (Pearson's correlation 0.0623, *p* < 0.05). The same result was obtained for all experiments (not shown). A comparison of the time to peak distributions (Kruskal–Wallis One Way Analysis of Variance on Ranks, with multiple comparisons performed using Tukey Test), showed a statistical difference (*p* < 0.05) between the experimental values and those obtained with synapses in the dendrites, and no difference between experiment and synapses near the soma. Taken together, these results suggest that all the fitted traces are from synaptic events elicited near the soma.

**Figure 7:**
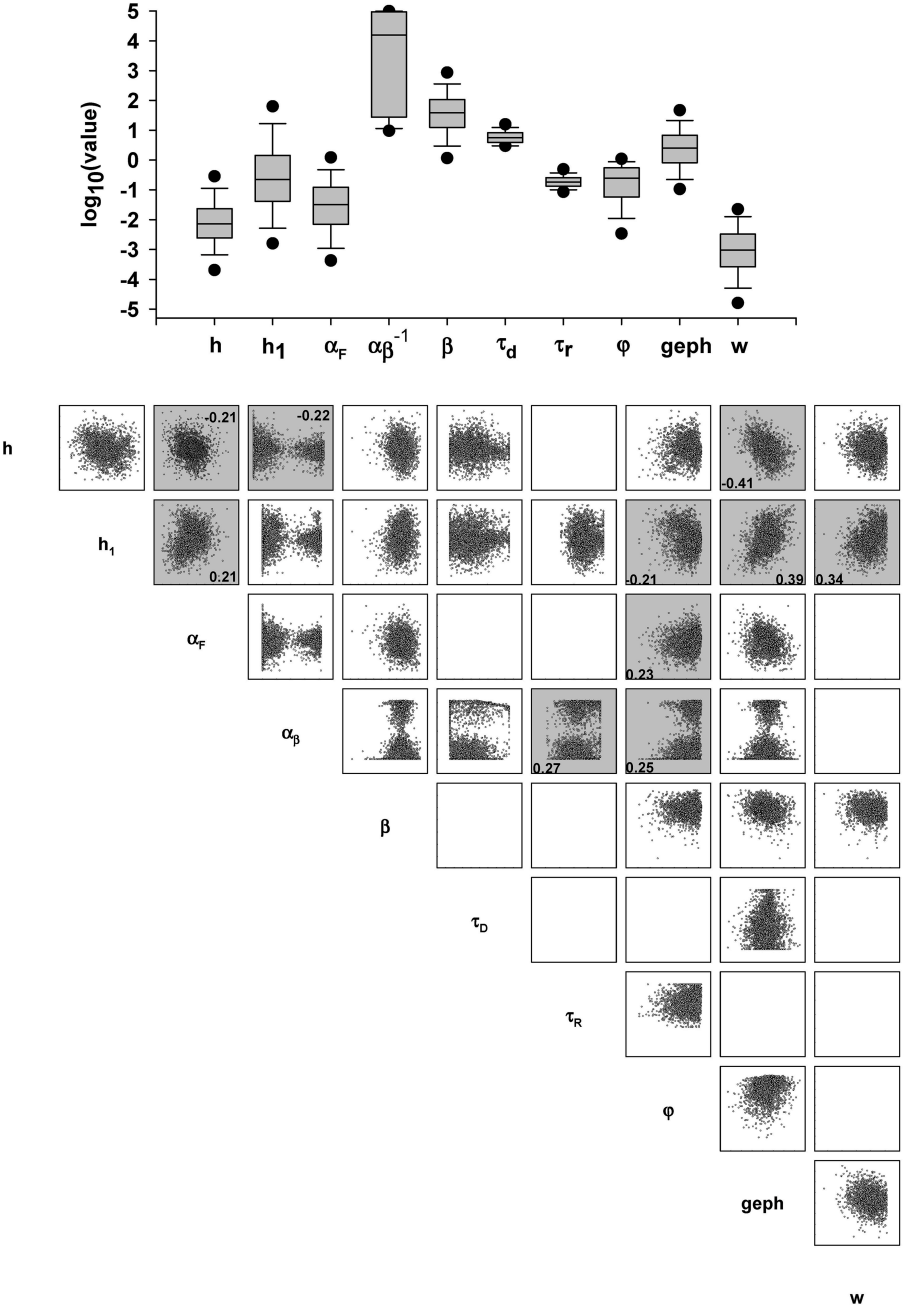
**Values and correlation of model parameters**. **Top:** Box plot of the parameter values obtained for the best fit for all traces; **Bottom:** correlation matrix; colored squares indicate an absolute value for Spearman's correlation coefficient above 0.2. The actual value is reported in the relative plot and, to highlight the correlation, points were plotted in log scale.

To assess the kind of information that can be obtained using our procedure on individual synaptic events, and how useful the information can be, we began with an analysis at the highest data level, pooling results from all experiments. The overall distribution of values obtained for each parameter is shown in Figure 7. The results reflect the wide range of allowed values for most parameters (as defined in the configuration file). The only exceptions were τ_*r*_ and τ_*d*_, constrained within a relatively narrow range of experimentally plausible values (0.05–0.5, ad 3–20 ms, respectively). To test for a possible correlation between parameters, a correlation matrix was calculated using the Spearman Rank Order Correlation (Figure 7, bottom). Many interesting and statistically significant positive or negative correlations were obtained between parameters. A closer look at the results, particularly those showing the highest correlation (Figure 7, plots with a gray background), reveals a relationship between specific parameters that can give useful information. For example, *h* was negatively correlated with α_*f*_, α_*b*_ and *GEPH*, whereas *h*_1_ was positively correlated with α_*f*_, *GEPH*, and the peak synaptic conductance *w*. These correlations very closely reflect the true relations among parameters, as can be seen from the analytical solution of the model equations (Equations 1, 2). This capability of the fitting procedure, to directly point out the relations among parameters, can be very useful when the model at hand is too complicated to be solved analytically.

**Figure 8:**
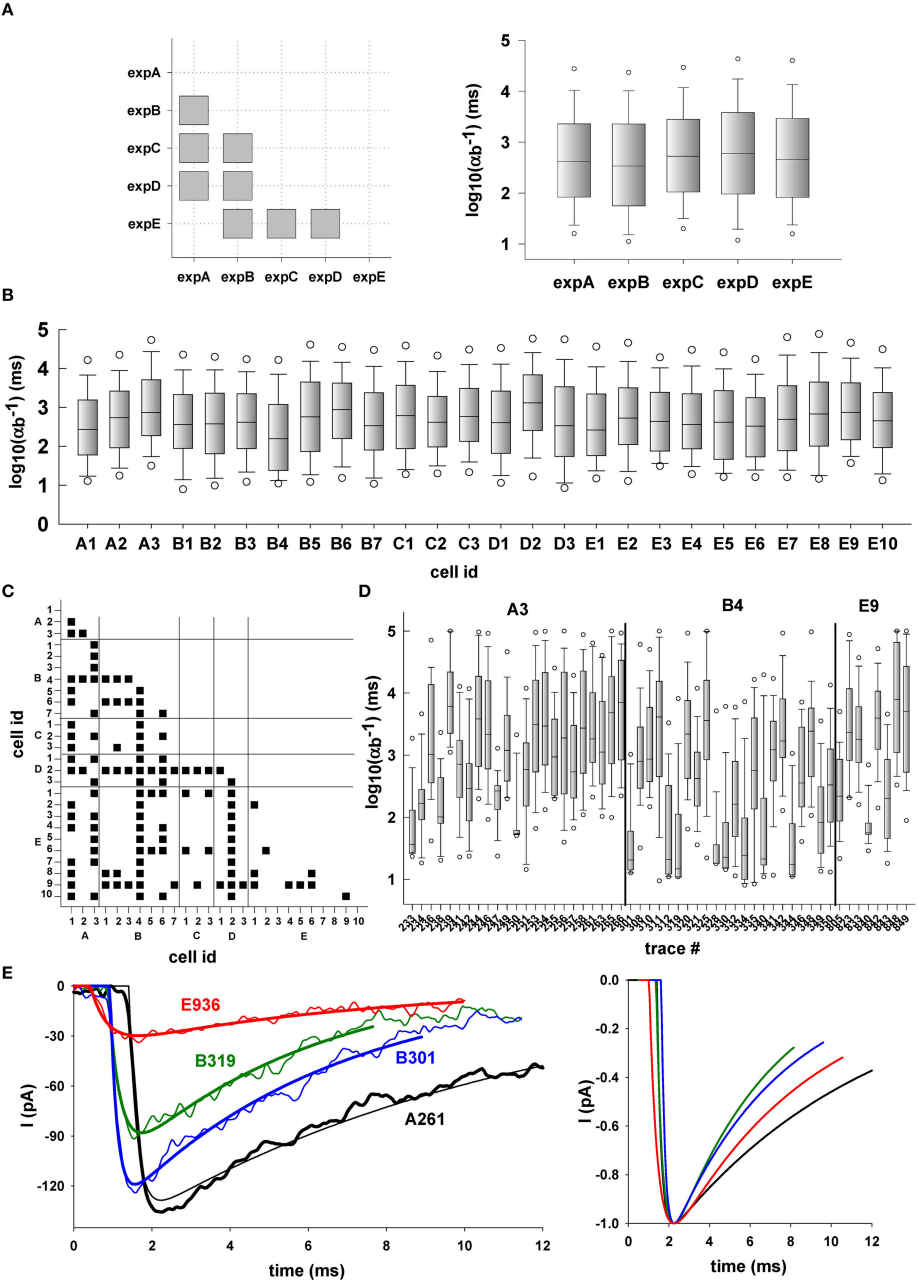
**Typical example of multilevel analysis of simulation results for α_*b*_. (A)** (Left) correlation at the experimental level; the plot shows cases (gray squares) in which there was a significant difference in the median values between values obtained by fitting traces from different experiments; (right) box plot of α_*b*_ values grouped by experiment; **(B)** Analysis at the single cell level; box plots of α_*b*_ values grouped by results obtained in recordings from the same cell; **(C)** Correlation at the single cell level; the plot shows cases (black squares) in which there was a significant difference between the median values obtained by fitting traces from different cells; **(D)** Analysis at the single trace level; selected examples of α_*b*_ values grouped by results obtained from multiple fits of the same trace; **(E)** Typical examples of traces (left, thin lines) and one of their fit (left, thick lines); (right) fitted traces are plotted aligned to their peak value to allow better comparison among different traces.

Next, we considered two example parameters (α_*b*_ and *I*_*FACT*_) that illustrate other type of results that can be obtained with our procedure. The first parameter, α_*b*_, represents the rate at which released neurotransmitter molecules diffuse away from the synaptic cleft, and is the main mechanism modulating extrasynaptic tonic inhibition, whereas *I*_*FACT*_ represents the time independent effects. In Figure 8 we show the distribution of α_*b*_ from different perspectives at different levels: *experimental*, to compare the results between different experiments; *cellular*, to compare the results from different neurons within the same experiment; and, *intracellular*, to compare the results for individual events recorded from the same neuron during a given experiment. Unless stated otherwise, we selected only the best fit for each trace, to minimize the possible bias caused by the many (good) fits of the less noisy traces. Unless otherwise noted, in all cases the parameter distributions were compared using Kruskal–Wallis One Way Analysis of Variance on Ranks, with multiple comparisons performed using Dunn's method.

Statistical comparison of the values for α_*b*_, grouped for each experiment (Figure 8A), indicates that the rate at which released neurotransmitter molecules diffuse away from the synaptic cleft can be significantly different between experiments (gray squares indicate *p* < 0.05). This was true in all cases, except for events during expA (juvenile C57Bl6/J male mice) and expE (3–4 months old female Tg2576 mice). The set of α_*b*_ values obtained in these two experiments were indistinguishable. In Figure 8B we show box plots of α_*b*_ grouped for each neuron in each experiment, whereas in Figure 8C we plot the correlation matrix comparing values obtained between neurons. Statistically significant differences are indicated with a black square, and they reveal information that could be used to better characterize the synaptic properties of specific neurons. For example, some neurons (e.g., A2, D3, and E10) have α_*b*_ values indistinguishable from those obtained for most of the other neurons, whereas other cells (e.g., B4 or D2) showed α_*b*_ values significantly different from all the other neurons, including those from the same experiment. Can these differences reflect specific synaptic configuration for specific neurons? To test this hypothesis, in Figure 8D, we grouped the α_*b*_ values obtained for a randomly chosen set of events from neurons A3, B4, and B9. Note that, as indicated in Figure 8C, A3 and E9 have the same distribution of α_*b*_ values, whereas that for B4 is different from both. As can be seen, events observed in cell B4 were preferentially fitted with α_*b*_ values lower than those from A3 and E9. This typically results in a faster decay for events recorded in B4, as shown by the typical traces (and their fit) in Figure 8E.

**Figure 9:**
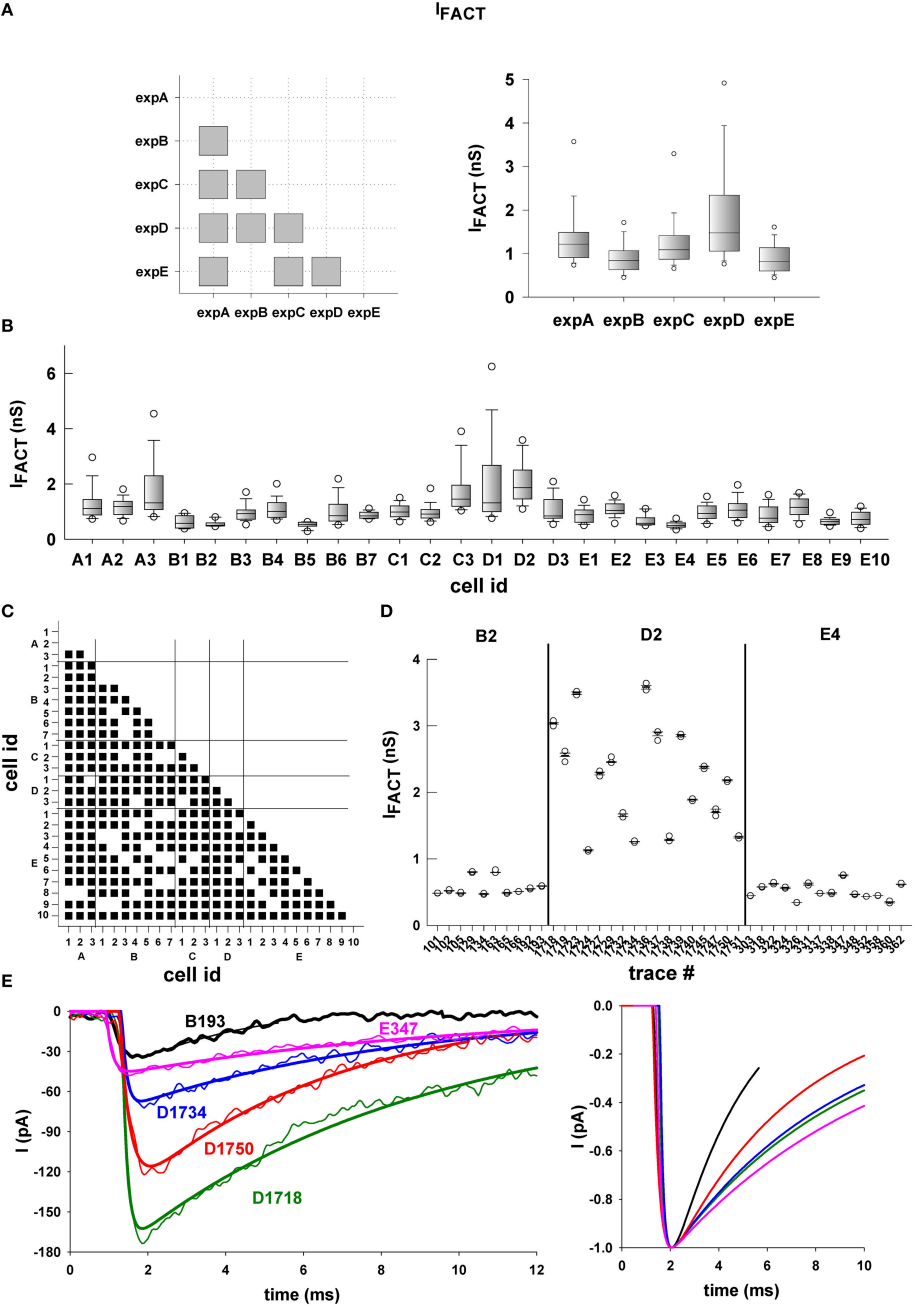
**Typical example of multilevel analysis of simulation results for *I*_*FACT*_**. Same as in Figure 8 but for *I*_*FACT*_.

When we performed the same analysis for *I*_*FACT*_ we again found a statistically significant difference between the set of values obtained in all experiments (i.e., the differences in the median values are greater than would be expected by chance). The only exception was between expE and expB (Figure 9A, left), which showed similar percentiles and median values (Figure 9A, right). In contrast with the results for α_*b*_, the overall range of values for *I*_*FACT*_ was much narrower, within one decade (Figure 9A, right). *I*_*FACT*_ distributions were rather different among cells (Figure 9B), and the set of values were statistically independent in almost all cases (Figure 9C). Typical examples for several traces from neurons B2, D2, and E4, are shown in Figure 9D, and a few of them are plotted in Figure 9E. Note that neurons B2 and E4 are among the few cases with similar *I*_*FACT*_ (see Figure 9C), and that expB and ExpE are from different mice species, with respect to expA, expC, and ExpD. A deeper analysis of the functional consequences for these results was outside the scope of this paper. However, taken together, these results show the amount, quality, and possible usefulness of the information that can be obtained using our procedure.

## Discussion

In this paper we present a general tool to study transsynaptic signal kinetics using *ex vivo* data from voltage-clamp recordings. The procedure will be available in ModelDB (acc.n. 182129), implemented using the NEURON simulation environment (Hines and Carnevale, 1997) and its built-in PRAXIS fitter. A Python version is installed, and available as a standalone task in the Collaboratory Portal of the Human Brain Project. The procedure can run in series or in parallel on conventional multiprocessor systems or supercomputers. Given the specific experimental preparation and protocol used (i.e., spontaneous inhibitory events recorded under somatic voltage clamp), the fitting does not require, in principle, any specific realistic biophysical implementation model of the cells (in this case hippocampal pyramidal CA1 neurons) from which the recordings were made.

The only limitation is that the events to be fitted need to be unaffected by space clamp errors. One of the possible problems in analyzing synaptic currents from somatic recordings is that, very often the traces obtained, and thus their fit, may not give an accurate representation of the transsynaptic signal dynamics. Inadequate voltage clamp of distant dendrites or the filtering properties of the membrane can significantly alter the time course of a synaptic current observed at the soma (Spruston et al., 1993). However, this may not be the case for GABAergic synapses, since a substantial proportion (at least 30%) are located in the proximal dendritic compartments (Megías et al., 2001). The issue may be particularly relevant for the fast components of the current, such as time to peak current. This is an important constraint, since the calculation of time to peak current requires the activation time and can only be determined by the fitting process. Our analysis of the time to peak obtained from the fits shows that the traces fitted by our procedure are not affected by space clamp or dendritic filtering issues; therefore, the results can be reliably used to study subcellular transsynaptic signaling pathways in inhibitory synapses.

From an initial set of experimental data and user-defined model kinetics, parameters, and rules, the procedure generates several suitable parameter ensembles fitting individual spontaneous synaptic events with an average RMSE < 10% of the peak value. External users can upload the experimental data and/or the model kinetics and configuration file, or choose among the models and dataset that will be made available in the future. Using the configuration file approach makes the procedure independent from the specific kinetic model, because the parameters and experimental protocol are defined in the configuration file. The same procedure could therefore be run several times using the same experimental data, using a different kinetic scheme and relative configuration file to investigate different specific pathways with different levels of detail. However, the overall quality of the fits crucially depends on the specific kinetic model used. The kind of information that can be gathered also depends on the specific kinetic scheme and experimental constraints on the fitting parameters. With a relatively simple and generic scheme such as that used in this work, the parameter space could be expected to have many basins of attraction, corresponding to several different parameter combinations that result in equally good fits. In our case, since we had an analytical solution for the dynamic system, we could even extract useful information on the synaptic events. To the best of our knowledge, this is the first, publicly available, procedure for fitting individual (rather than averaged) synaptic events with any user-defined kinetic model and a non-convex optimization. We think that this approach can give useful information on the possible correlation between parameters in reproducing the observed events, and eventually help to improve the kinetic model with a better representation specific synaptic transmission mechanisms. The use of a non-convex optimization method also adds an additional source of information on the underlying mechanisms. By analyzing the many possible ways to obtain the same trace with different parameter combinations, one can extract useful indications, for example, on the model limitations or on the relative contribution of each parameter on the overall trace. This kind of analysis depends, of course, on the specific kinetic model used for the fitting procedure.

The results can be used/analyzed according to the user's plan of investigating specific neuronal systems, or carrying out simulations using the parameter ensembles to generate a population of synaptic inputs reproducing a statistically realistic circuit configuration. Working with parameters representing individual synaptic events is an important step in creating simulations of biophysically accurate neuron and network models. From this point of view, we have shown how the choice to fit and analyze individual events, rather than average traces, can lead to interesting comparisons and conclusions for the events recorded from different experimental protocols and conditions. Indeed, the wide range of values that can be obtained experimentally, an example of which can be seen in Figure 2 from our experimental data, stress the importance of fitting individual events rather than an average. In this paper we chose to implement a relatively simple model for gephyrin, one of the major players in the functional organization of inhibitory synapse. One of the reasons for this choice is that we wanted to test our procedure against experimental recordings of GABAergic events, and there are currently no computational models available for gephyrin.

Although, as explicitly mentioned in Methods, we could have used any kinetic scheme including a biochemical pathway involving at least one of the tens of thousands of proteins present in a synapse, we think that the choice to model a basic gephyrin-dependent pathway turned out to be a useful application of the procedure. The overall direct and indirect roles of gephyrin as synaptic scaffolding mechanism appear to be clear, but its functional modulation of the transsynaptic signal, and the interaction with other subcellular pathways, is less clear. Gephyrin is a tubulin-binding protein that, through its self-oligomerizing properties, forms hexagonal lattices that trap GABA_A_ receptors in the right place at postsynaptic sites by linking them to the cytoskeleton (Tretter et al., 2008). In addition, gephyrin contributes to backward control on presynaptic signaling *via* specialized adhesion molecules such as NLGs and NRXNs which, by bridging the cleft, provide a direct link between the post and the presynaptic sites (Lisé and El-Husseini, 2006). Disrupting endogenous gephyrin with selective intrabodies (scFv-gephyrin), acting at post-translational level (Zacchi et al., 2008), leads to a reduction of GABA_A_ receptor clusters, a decrease in the density and size of NLG-2 clusters and loss of GABAergic innervation (Marchionni et al., 2009; Varley et al., 2011). The model findings suggest how the transsynaptic signal can be strongly dependent not only on the gephyrin level, but also on the turnover rate of NLG/NRXN, and the extrasynaptic diffusion of neurotransmitter molecules. Small changes of these mechanisms can have large effects on the synaptic current, and these can be cell- or event-specific, with individual events that can be correlated with functional or pathological aspects at different levels. The interesting significant correlations that were noted (Figure 7) reflect the structure of a dynamic system, suggesting, for example, that the mechanisms involved during a given synaptic event may be determined by a high number of receptors (controlled by gephyrin) and a low level of neurotransmitter molecules (modulated by NLG2). In the simple case used in this procedure, the analytical solution gives a relatively clear and complete picture of the underlying mechanisms. However, more detailed implementations of the transsynaptic subcellular pathways cannot be solved analytically, so the type of procedure outlined here may help identify critical subcellular pathways.

Our results indicate that the rate at which neurotransmitters diffuse away from the synaptic cleft (α_*b*_, Figure 8A) can be significantly different for each experiment. Considering the wide distribution observed at this investigation level (Figure 8A, right), this result was rather surprising. We would have expected many combination of parameters resulting in the same good fit with widely different values of α_*b*_ for each event. The fact that some neurons have very similar α_*b*_ values, while other have very different values (Figure 8C), suggests that there may be neuron-specific mechanisms regulating the extrasynaptic diffusion of GABA. These differences were shown by analysis at the individual event level, carried out using all acceptable fits for each trace (i.e., RMSE < 10% of the peak). A high difference among different events was observed, even if recorded from the same neuron. In general, a qualitative examination of the data in Figure 8D suggests that each cell may have groups of synapses with similar α_*b*_. This may explain why the distribution of α_*b*_ values for events in cell A3 is significantly different from events in cell B4 but not significantly different from those in cell E9. The differences may merely reflect physiological variability in the transsynaptic dynamics or specific differences in the underlying mechanisms. We also found a similar lack of statistical correlation for the values of *I*_*FACT*_ in all the experiments, with the exception of expE and expB, where we observed a similar distribution of values. It should be noted that *I*_*FACT*_ is a combination of those parameters that directly coincide to determine the peak value of the current; it therefore most likely reflects the effect of synaptic plasticity mechanisms activated by the history of a synaptic input. The results suggest that they can be cell- or event-specific, with individual events that can be correlated with functional or pathological aspects at different levels.

We have therefore shown that our kinetic model for fitting individual synaptic events from voltage clamp experiments may be used reliably to study subcellular transsynaptic signaling pathways in inhibitory synapses, using gephyrin as an example. For individual spontaneous inhibitory events in hippocampal pyramidal CA1 neurons, we found that gephyrin-dependent subcellular pathways may shape synaptic events at different levels, and can be correlated with cell- or event-specific activity history and/or pathological conditions. The functional consequences of interfering with gephyrin-dependent mechanisms at a higher level, for example in terms of the I/O properties of a hippocampal CA1 pyramidal neuron, will be explored in a future paper.

## Author contributions

CL, substantial contributions to numerical simulation and data analysis. AM, EM, SM, CM, and EC substantial contributions to the experiments. RM substantial contributions to the design and analysis of the theoretical scheme. MM, substantial contributions to the conception and design of the work, to the analysis and interpretation of data for the work; drafting the work. All the authors revised the work critically for important intellectual content, approved the final version and agreed to be accountable for all aspects of the work in ensuring that questions related to the accuracy or integrity of any part of the work are appropriately investigated and resolved.

### Conflict of interest statement

The authors declare that the research was conducted in the absence of any commercial or financial relationships that could be construed as a potential conflict of interest.
